# PM_2.5_ obtained from urban areas in Beijing induces apoptosis by activating nuclear factor-kappa B

**DOI:** 10.1186/s40779-017-0136-3

**Published:** 2017-08-31

**Authors:** Hui Peng, Xiao-Hong Zhao, Ting-Ting Bi, Xiao-Yan Yuan, Jia-Bin Guo, Shuang-Qing Peng

**Affiliations:** 1 Evaluation and Research Center for Toxicology, Institute of Disease Control and Prevention, Academy of Military Medical Sciences, Beijing, 100071 China; 2 Beijing Key Laboratory of Bioactive Substances and Functional Foods, Beijing Union University, Beijing, 100191 China

**Keywords:** Particulate matter, NF-κB pathway, Apoptosis, BAD protein

## Abstract

**Background:**

Particulate matter (PM), which has adverse effects on citizen health, is a major air pollutant in Beijing city. PM_2.5_ is an indicator of PM in urban areas and can cause serious damage to human health. Many epidemiological studies have shown that nuclear factor-kappa B (NF-κB) is involved in PM_2.5_-induced cell injury, but the exact mechanisms are not well understood.

**Methods:**

The cytotoxic effects of PM_2.5_ at 25–1600 μg/ml for 24 h were determined by MTT assay in Chinese hamster ovary cells (CHO) cells. Flow cytometry was used to determine the apoptosis rate induced by PM_2.5_. The destabilized enhanced green fluorescent protein (d2EGFP) green fluorescent protein reporter system was used to determine the NF-κB activity induced by PM_2.5_. The expression of pro-apoptotic Bcl-2-associated death promoter (BAD) proteins induced by PM_2.5_ was determined by western blotting to explore the relationship between PM_2.5_ and the NF-κB signaling pathway and to determine the toxicological mechanisms of PM_2.5_.

**Results:**

PM_2.5_ collected in Beijing urban districts induces cytotoxic effects in CHO cells according to MTT assay with 72.28% cell viability rates even at 200 μg/ml PM_2.5_ and flow cytometry assays with 26.97% apoptosis rates at 200 μg/ml PM_2.5_. PM_2.5_ increases the activation levels of NF-κB, which have maintained for 24 h. 200 μg/ml PM_2.5_ cause activation of NF-κB after exposure for 4 h, the activation peak appears after 13.5 h with a peak value of 25.41%. The average percentage of NF-κB activation in whole 24 h is up to 12.9% by 200 μg/ml PM_2.5_. In addition, PM_2.5_ decreases the expression level of the pro-apoptotic protein BAD in a concentration-dependent manner.

**Conclusions:**

PM_2.5_ induces NF-κB activation, which persists for 24 h. The expression of pro-apoptotic protein BAD decreased with increased concentrations of PM_2.5_. These findings suggest that PM_2.5_ plays a major role in apoptosis by activating the NF-κB signaling pathway and reducing BAD protein expression.

## Background

Urban air particulate matter (PM), which is a major atmospheric pollutant, is correlated with many adverse health effects, including increased respiratory and cardiovascular morbidity and mortality [1–3]. Recent epidemiological studies have recognized PM_2.5_ (particulate matter with an aerodynamic diameter ≤ 2.5 μm) as an important indicator of fine particulates [4, 5]. Urban air PM_2.5_ consists primarily of soot, which has different components, including organic elements, metals, and biological contaminants, and it is produced by the combustion of fossil fuel and vehicle exhaust. These fine particles easily reach the distal regions of the lungs and are retained in the alveolar walls, which results in allergies, asthma, and lung emphysema [6–8]. PM_2.5_ is a major pollutant in Beijing urban districts, where the annual mean PM_2.5_ concentration (90.7 μg/m^3^ average in 2013) exceeds the WHO air quality guidelines for PM of an annual mean of 10 μg/m^3^ [9, 10].

Although significant efforts have been made to understand the lung damage induced by PM_2.5_ [5, 11, 12], the underlying mechanisms by which PM_2.5_ induces adverse health effects are still unclear. There is evidence that PM_2.5_ induces cell apoptosis in lung cells [13, 14]. Apoptosis regulates the cell cycle and plays a critical role in both tissue homeostasis and the development of various diseases. Previous studies have demonstrated that nuclear factor-kappa B (NF-κB) is a major anti-apoptosis transcription factor that is vital for immune responses, inflammation, and cell survival [15–17] and that it plays an important role in the apoptotic effect of PM_2.5_ [18, 19].

Many epidemiological studies have shown that the NF-κB signaling pathway plays an important role in lung injury from inhaled particle matter [20–23]. The NF-κB family includes five members (RelA/p65, RelB/p68, cRel/p75, p52, and p50), and NF-κB classically refers to the p65:p50 complex. NF-κB activity is regulated by cytoplasmic degradation of the IκB (inhibitor of κB) inhibitor. Once IκBα is inactivated, NF-κB dimers localize to the nucleus and undergo further modification, which mostly occurs via the phosphorylation of the Rel proteins. In the nucleus, activated NF-κB binds to promoters of its target genes and regulates their expression, including the activity of anti-apoptotic genes, such as Bcl-2 family members (e.g., Bcl-2, Bcl-xl) [24, 25].

The Bcl-2-associated death promoter (BAD) protein has been shown to dimerize with anti-apoptotic proteins Bcl-2 and Bcl-xl. BAD is a member of the BH_3_-only family, which is a subfamily of the Bcl-2 family [26]. When BAD is phosphorylated by Akt/protein kinase B, which is an important upstream element of the NF-κB pathway, it forms the BAD-(14–3-3) protein heterodimer. This change leaves Bcl-2 free to inhibit Bax-triggered apoptosis [27]. BAD phosphorylation is therefore anti-apoptotic, and BAD dephosphorylation is pro-apoptotic. BAD is a promoter of apoptosis in the Bcl-2 family that promotes apoptosis and interacts with caspases [28, 29]. However, the exact mechanism by which the NF-κB acts in PM-induced cell apoptosis is poorly understood.

In this study, we investigated cell proliferation, apoptosis, activation of NF-κB, and the expression of pro-apoptotic protein-BAD proteins induced by PM_2.5_ to explore the relationship between PM_2.5_ and the NF-κB signaling pathway to elucidate the toxicological mechanisms of PM_2.5_.

## Methods

### Reagents

F12-K nutrient mixture (Kaighn’s modification) and fetal bovine serum (FBS) were purchased from GIBCO Co. (NJ, USA). Glutamine, penicillin, streptomycin and Lipofectamine™ 2000 were purchased from Invitrogen (CA, USA). Quartz filters (203 × 254 mm) were purchased from Whatman Co. (PA, USA). Hoechst 33342 dye, thiazolyl blue tetrazolium bromide (MTT), pyrrolidine dithiocarbamate (PDTC) and lipopolysaccharide (LPS) (*E coli*, serotype 055:B5) were purchased from Sigma Chemical Co. (MO, USA). The Annexin V-FITC apoptosis detection kit was purchased from Biosea Biotechnology Co. (Beijing, China). PVDF membranes were purchased from Millipore Co. (MA, USA). Anti-β-actin, anti-Bad (19–35) rabbit pAb were purchased from MBL (MA, USA). The Phototope®-HRP western blot detection system was purchased from Cell Signaling Co. (MA, USA). Western stripping buffer was purchased from the Beyotime institute of biotechnology (Beijing, China). The Cell and tissue protein extraction reagent, protease inhibitor cocktail, phosphotase inhibitor cocktail and phenylmethylsulfonyl fluoride (PMSF) were purchased from KangChen Biotech Co (Beijing, China). All other chemicals were analytical grade and were purchased from Sigma Chemical Co. or DingGuo Biochemical Co. (Beijing, China).

### Cell culture and vectors

The Chinese hamster ovary (CHO) cells were purchased from Shanghai Institutes for Biological Sciences, Chinese Academy of Sciences, and were cultured in a 75 cm^2^ flask in F12-K medium supplemented with 10% heat-inactivated fetal bovine serum (FBS), 2 mmol/L glutamine, penicillin (100 units/ml) and streptomycin sulfate (100 mg/ml) at 37 °C in a humidified incubator containing 5% CO_2_. At 90% confluency, cells were harvested using 0.25% trypsin and were sub-cultured in a 75 cm^2^ flask, 24-well plates or 96-well plates. The vector pNF-κB-d2EGFP was purchased from Clontech (CA, USA) and used to monitor the activation of the NF-κB signal transduction pathway. Induction of the pathway enables endogenous NF-κB to bind to the kappa (κ) enhancer element (KB_4_, 21–67 sites, 5′-GGGAATTTCC-3′) located in the promoter region of the vector and the d2EGFP (destabilized enhanced green fluorescent protein) coding sequence was followed by the SV40 late polyadenylation signal.

### PM_2.5_ collection and preparation

PM_2.5_ samples were collected into quartz filters using the TH-1000 TSP high volume sampler (Tianhong Instrument Co., Ltd., Wuhan, China) from Dec 24, 2008 to Jan 16, 2009 in Xueyuan Road, Beijing City. The PM_2.5_ sampler was placed on the rooftop of a building approximately 9 meter tall beside Xueyuan Road, and there were no large obstacles near the building. The sampler was controlled at a flow rate of 78 m^3^/h for a period of 22 h, with a 2-h interval. PM_2.5_ was extracted as previously described with minor modifications [30]. The sampled filters were cut into small pieces of 6 cm^2^ and then put into a sterilized beaker with 50 ml sterilized pure water; sonicated for 1 min in water (below 25 °C) and then filtrated with 6-fold sterilized gauze to obtain a PM_2.5_ suspension. PM_2.5_ suspensions were freeze-dried in vacuum for 24 h and stored at −20 °C prior to use, the dried samples were diluted with sterilized phosphate-buffered saline solution (PBS, pH 7.2) and sonicated for 15 min to disperse the possible aggregates.

### Cell viability assay

The cytotoxic effects of PM_2.5_ were determined by an MTT assay. Briefly, CHO cells were cultured in 24-well plates at a density of 5.0 × 10^4^ cell/ml and incubated overnight. The medium was then changed, and the cells were incubated with a suspension of PM_2.5_ (25–1600 μg/ml) for 24 h and added to MTT solution at final concentration (0.5 mg/ml) for 4 h and then to 200 μl DMSO (dimethyl sulfoxide) for 15 min. The reaction mixture in a 24-well plate was transferred to a 96-well plate with 6 parallel samples. Cell viability was estimated by measuring the OD value using the μQuant Microplate Spectrophotometer MQX200 (Bio-Tek, GA, USA) at a wavelength of 570 nm and was normalized to treated cells in medium only (100% viability). The experiments were repeated three times.

### Measurement of apoptosis

Flow cytometry was used to determine the apoptosis rate induced by PM_2.5_. Briefly, CHO cells were seeded in 6-well plates at 5.0 × 10^4^/ml in 2 ml F12-K medium supplemented with 10% FBS and incubated overnight. The medium was changed, and the cells were treated with different concentrations of PM_2.5_ for 24 h. Then, the cells were trypsinized, pelleted, and resuspended at 5 × 10^5^ cells/ml in ice-cold PBS. Three volumes of ice-cold 70% ethanol were added to the mixture gradually and gently shaken. The cell suspension was pelleted and resuspended in 300 μl binding buffer, and then 10 μl Annexin V-Cy5 and 5 μl PI were added. Stained cells were incubated for 30 min with protection from light. Next, 200 μl of binding buffer was added before analysis. The samples were measured with a FACScan (Becton-Dickinson, NJ, USA) flow cytometer. For each sample, 1 × 10^4^ cells were analyzed. The data were collected for each analysis. The measurement of apoptosis was all types of apoptosis, including early apoptosis, late apoptosis and necrosis.

### Construct NF-κB activation responsive d2EGFP reporter system

The pNF-κB-d2EGFP vector, which contained a d2EGFP reporter gene and 4 copies of NF-κB *cis*-element κB, was used to construct an ideal NF-κB activation responsive d2EGFP reporter system. CHO cells were seeded into 96-well plates at 4.0 × 10^4^/ml in 200 μl of F12-K medium supplemented with 10% FBS without antibiotics and incubated for 18 h. After 1 h of incubation, the cells were transfected by Lipofectamine™ 2000 reagent with the plasmid pNF-κB-d2EGFP. After 4 h of incubation, cells were stained with 10 μg/ml Hoechst 33342 dye for 15 min and treated with different test samples. LPS was selected as an activator of NF-κB and PDTC was selected as an inhibitor of NF-κB that could inhibit the activation of NF-κB induced by LPS.

### Measurement of NF-κB activity

With Hoechst 33342 dye staining and the fluorescence of d2EGFP, a live cell image analysis system-IN Cell Analyzer 1000 system (GE Healthcare, PA, USA) was used to obtain the cell images. The plate was put on the plate holder of an IN Cell Analyzer 1000 system and the environmental control module was adjusted to the same conditions as a CO_2_ incubator. The images of CHO cells were acquired every 30 min for 24 h. The plasma membrane spot analysis module was used to determine the level of d2EGFP signal (NF-κB expression) throughout the cell and then identified and quantified the fluorescence intensity of the cell. The activity of NF-κB was represented by the percentage of the cells containing green fluorescence in the counted cells. The NF-κB activation data were collected by the IN Cell Analyzer 1000 every half an hour.

### Measurement of BAD protein expression

In short, CHO cells were seeded in 6-well plates at 5.0 × 10^4^/well in 2 ml of F12-K medium supplemented with 10% FBS and incubated overnight. The medium was then changed, and the cells were treated with test samples for 24 h. Isolation of cell fractions and western blotting were performed as described by Tang [31] and Woods et al. [32]. The blots were incubated with a rabbit anti-hamster BAD primary antibody (1:1000) and a HRP-conjugated mouse anti-rabbit secondary antibody (1:10,000). Protein bands on the membrane were detected using the Phototope®-HRP western blot detection system and exposed on high-performance chemiluminescence film (Kodak). Western stripping buffer was used to wash the bands and detect the expression of the β-actin protein. The bands were quantified by an ImageQuant RT ECL imaging system and the ImageQuant TL Plus 7.0 software (GE Healthcare, PA, USA).

### Statistical analysis

All data were presented as the mean ± standard deviation (SD) for each group and analyzed using the Statistical Package for the Social Sciences (SPSS) version 15.0. The presence of significant differences among groups was determined by ANOVA and Least Significant Difference (LSD) was used to compare the effects between each PM_2.5_ exposed group and the control group (significance *P* < 0.05 or 0.01).

## Results

### Effects of PM_2.5_ on cell viability

**Fig. 1:**
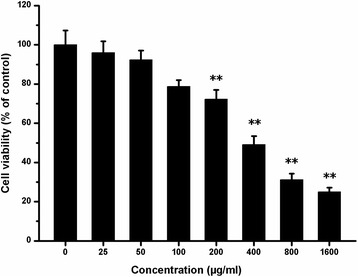
Effects of PM_2.5_ on cell viability. CHO cells were cultured in 24-well plates at a density of 5.0 × 10^4^ cells/ml in 0.5 ml of F12-K medium supplemented with 10% FBS and incubated overnight. The medium was changed, and the cells were treated with PM_2.5_ for 24 h. After 24 h, cell proliferation was measured by MTT assay. The data are expressed as the mean ± SD. Each data point represents an average of 12 samples. Experimental samples are compared with the untreated control, **P < 0.01

CHO cells were incubated for 24 h in the presence of PM_2.5_ at various concentrations. Cell viability was measured by MTT assay. The results are shown in Fig. 1. Cell viability rates were 96.24, 92.38, 78.78, 72.28, 49.07, 31.17, and 25.09% at 25, 50, 100, 200, 400, 800, and 1600 μg/ml of PM_2.5_, respectively, compared to the untreated control group (*P* < 0.01). The results show that PM_2.5_ inhibits cell viability in a dose-dependent manner (*r* = −0.962, *P* < 0.01).

### Induction of apoptosis by PM_2.5_ in CHO

**Fig. 2:**
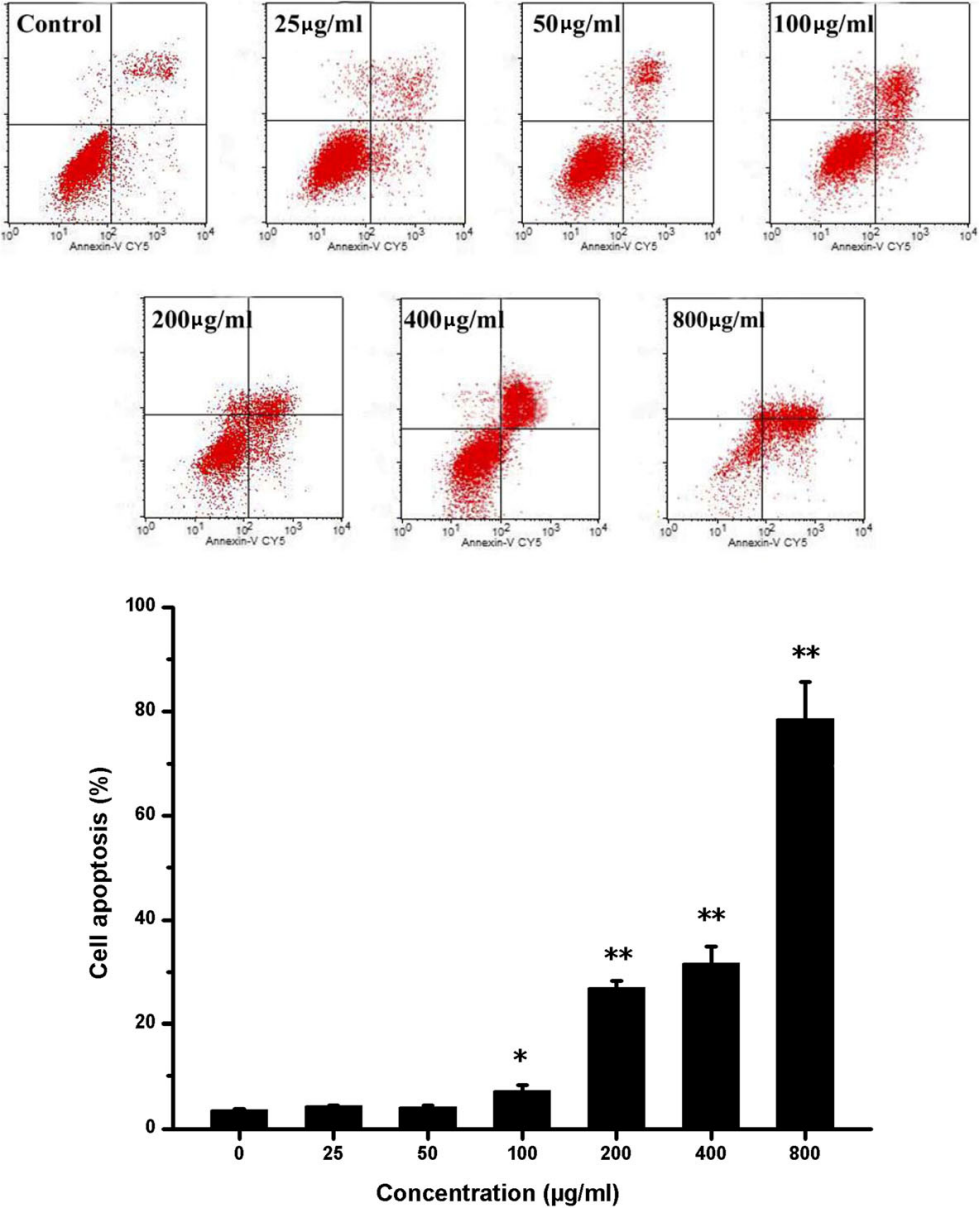
Effects of PM_2.5_ on cell apoptosis. CHO cells were cultured in 6-well plates at a density of 5.0 × 10^4^ cells/ml in 2 ml of F12-K medium supplemented with 10% FBS and incubated overnight. The medium was changed, and the cells were treated with different doses of PM_2.5_ for 24 h. After 24 h, cell apoptosis was measured by flow cytometry. Data are expressed as the mean ± SD. Each data point represents an average of 3 samples. Experimental samples are compared with the untreated control, *P < 0.05, **P < 0.01

To gain further insight into the mechanism of PM_2.5_-inhibited cell proliferation, we examined apoptosis after treatment with PM_2.5_ at concentrations from 25 to 800 μg/ml. The apoptosis rate of CHO cells was calculated and is shown in Fig. 2. The cell apoptosis rates increased as the concentration of PM_2.5_ increased. The apoptosis rates were 7.18, 26.97, 31.66, and 78.50% at 100, 200, 400, and 800 μg/ml PM_2.5_, respectively. Compared to the untreated control group, there were significant increases (*P* < 0.05 or *P* < 0.01).

### Effects of PM_2.5_ on NF-κB activity

**Fig. 3:**
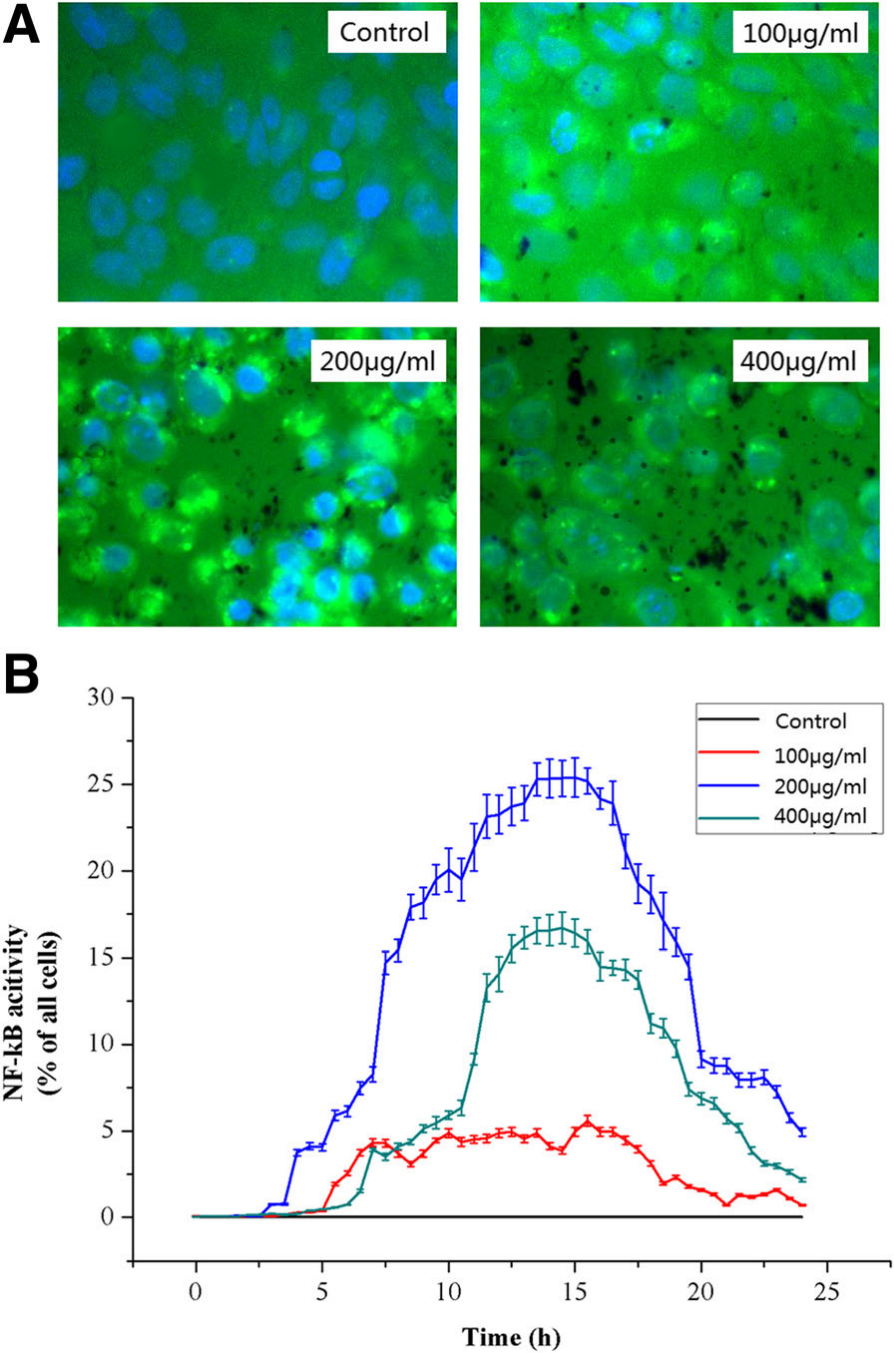
Effects of PM_2.5_ on NF-κB activity in CHO cells. **a**. The fluorescent cell images (NF-κB activation) obtained from an IN Cell Analyzer 1000 system. The nucleus is shown in blue and the cytoplasm in green. **b**. The effects of PM_2.5_ on NF-κB activity in the NF-κB activation responsive d2EGFP reporter system. CHO cells were seeded on 96-well plates at 4.0 × 10^4^ cells/ml in 200 μl F12-K medium supplemented with 10% FBS without antibiotics and incubated for 18 h until the cells reached 90–95% confluency. The cells were transfected and treated with 100, 200, and 400 μg/ml PM_2.5_. An IN Cell Analyzer 1000 system was used to obtain and analyze images of the cells every half an hour. Data are expressed as the mean ± SD. Each data point represents an average of 3 samples

The NF-κB activation responsive d2EGFP reporter system was used to investigate the effects that PM_2.5_ has on the dynamic activation of the NF-κB pathway. The images of fluorescence CHO cells obtained by an IN Cell Analyzer 1000 system are shown in Fig. 3a. In these images, PM_2.5_ is shown in black, and the nucleus is in blue due to staining with Hoechst 33342 dye. The cytoplasm is not seen in the resting cells when the NF-κB pathway is not activated. If the NF-κB is activated in the cell, the cytoplasm is green due to the green fluorescent protein. The concentrations of PM_2.5_ that were used were 100, 200, and 400 μg/ml. The green fluorescence in the PM_2.5_ treated groups represents the activated NF-κB by PM_2.5_.

PM_2.5_ increases the activation levels of NF-κB in the NF-κB-responsive d2EGFP reporter system, as shown in Fig. 3b. When exposed to 100 μg/ml PM_2.5_, activation occurred after 5.5 h and lasted approximately 16 h without a significant activation peak. However, 200 μg/ml PM_2.5_ caused NF-κB activation after 4 h; the activation peak appeared after 13.5 h with a peak value of 25.41%, and it continued for 2 h. After 22 h, NF-κB activation disappeared. In the presence of 400 μg/ml PM_2.5_, activation occurred after 13.5 h, with a peak value of 16.71%, continued for 3 h, and disappeared after 20 h.

### Effects of 200 μg/ml PM_2.5_ on NF-κB activity with an activator (LPS) and inhibitor (PDTC)

**Fig. 4:**
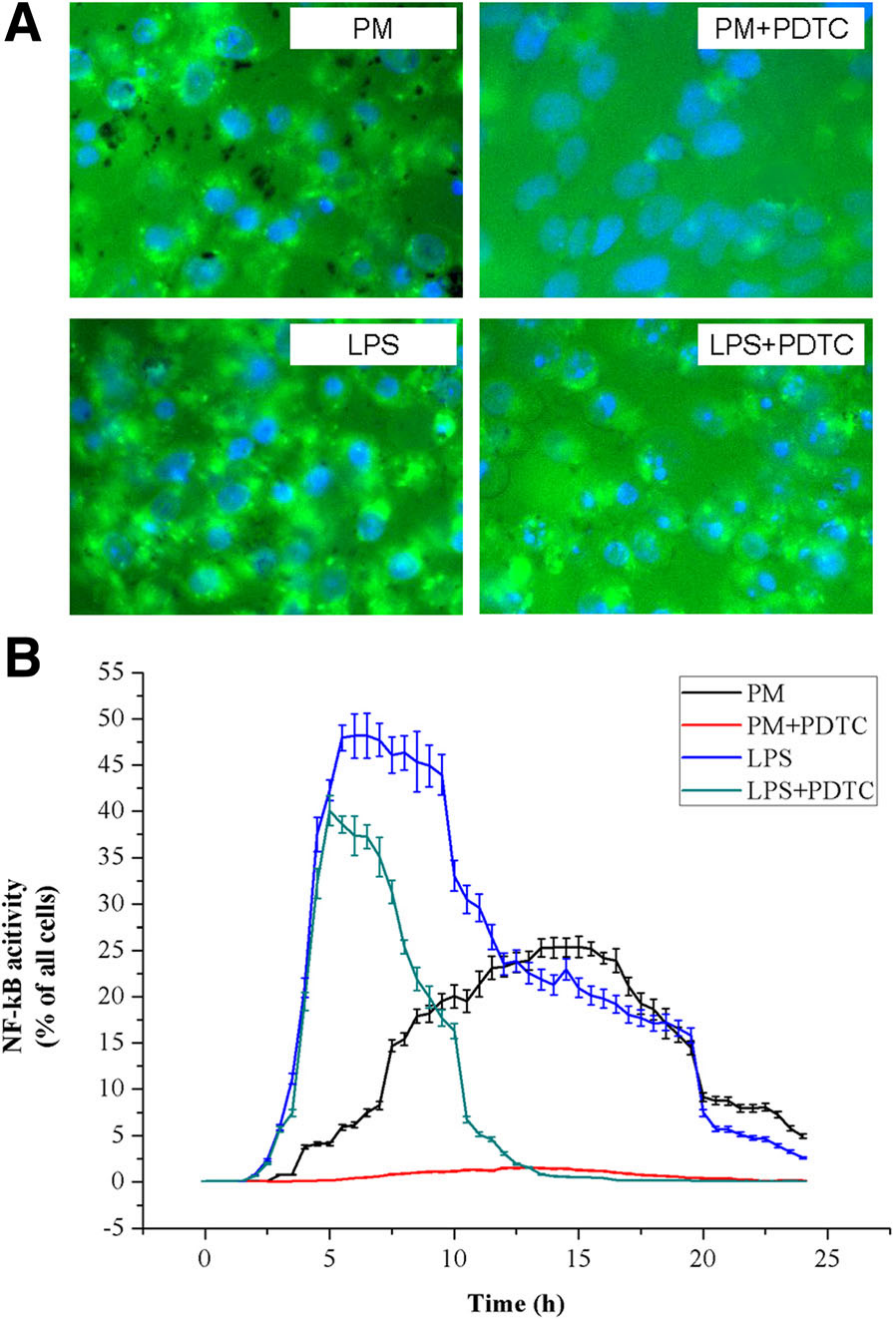
Effects of 200 μg/ml PM_2.5_ on NF-κB activity with an activator (LPS) and inhibitor (PDTC). **a**. Fluorescent cells. **b**. The effects of LPS and PDTC on NF-κB activity. CHO cells were treated with 200 μg/ml PM_2.5_. Cells in the PM + PDTC group were treated with 200 μg/ml PM_2.5_ and 100 μmol/L PDTC. Cells in the LPS group were treated with 100 ng/ml LPS for 2 h and switched to F12-K medium without serum. Cells in the LPS + PDTC group were treated with 100 ng/ml LPS for 2 h and changed to medium containing 100 μmol/L PDTC. An IN Cell Analyzer 1000 system was used to obtain and analyze images of cells every half an hour. The negative control is the same control used in Fig. 3 with the same curve data. The data are expressed as the mean ± SD. Each data point represents an average of three samples

CHO cells were treated with 200 μg/ml PM_2.5_. Cells in the PM + PDTC group were treated with both 200 μg/ml PM_2.5_ and 100 μmol/L PDTC. Cells in the LPS group were treated with 100 ng/ml LPS for 2 h and changed to F12-K medium without serum. Cells in the LPS + PDTC group were treated with 100 ng/ml LPS for 2 h and switched to medium containing 100 μmol/L PDTC. As shown in Fig. 4a, the green fluorescent signal is seen in the LPS and LPS + PDTC groups, which indicates that NF-κB is activated by LPS even in the presence of the inhibitor PDTC.

As shown in Fig. 4b, LPS significantly elevates the activity of NF-κB. Activation occurred after 2 h, reached a peak value of 49.19% after 5.5 h, continued for 2 h, and disappeared after 22 h. The activation of NF-κB by LPS is inhibited by PDTC. PDTC inhibited the activation of NF-κB induced by 200 μg/ml PM_2.5_, and the peak value decreased from 25.41 to 1.52%. These results indicate that LPS alters the peak activation time of PM_2.5_ and that the NF-κB-responsive d2EGFP reporter system detects NF-κB activation effectively, accurately, and dynamically.

The average percentages of NF-κB activation, shown in Table 1 **,** were calculated using the percentage of activated cells of the total number of cells over the whole 24 h. Compared to the untreated control group, the averages of the NF-κB activation ratio in the PM_2.5_-treated groups at concentrations ranging from 100 to 400 μg/ml significantly increased (*P* < 0.01). Compared to the 200 μg/ml PM_2.5_ group, the average NF-κB activation percentage of the 200 μg/ml PM_2.5_ and 100 μmol PTCD group significantly decreased (*P* < 0.01). Compared to the LPS group, the average NF-κB activation percentage of the 200 μg/ml PM_2.5_ group significantly decreased (*P* < 0.01).

**Table 1:** Effects of PM_2.5_ on NF-κB activity in CHO cells (x ± SD, *n* = 3)

Treatments	Activation percentage (%)
Control	0.007 ± 0.001
PM_2.5_ 100 μg/ml	2.606 ± 0.259^**^
PM_2.5_ 200 μg/ml	12.899 ± 0.385^**^
PM_2.5_ 400 μg/ml	6.717 ± 0.312^*​*^
PM_2.5_ 200 μg/ml + PDTC 100 μmol/L	0.634 ± 0.028^##^
LPS 100 ng/ml	20.836 ± 1.265^**^
LPS 100 ng/ml + PDTC 100 μmol/L	8.497 ± 0.962^ΔΔ^

Compared with the untreated control, ^**^ *P* < 0.01; compared with PM2.5 200 μg/ml, ^##^ *P* < 0.01; compared with LPS, ^ΔΔ^ *P* < 0.01

 ﻿CHO cells were seeded on 96-well plates at a density of 4.0 × 10^4^ cells/ml in 200 μl of F12-K medium supplemented with 10% FBS without antibiotics and incubated for 18 h until the cells reached 90–95% confluency. The cells were transfected and treated with test samples. An IN Cell Analyzer 1000 system was used to obtain and analyze images of cells every half an hour. The average percentage of NF-κB activation was calculated by the percentage of activated cells of all cells over the whole 24 h. Data are expressed as the mean ± SD. Each data point represents an average of 3 samples. Compared with the control, ^**^ *P* < 0.01; compared with PM_2.5_ 200 μg/ml, ^##^ *P* < 0.01; compared with LPS, ^ΔΔ^ *P* < 0.01.

### Effects of PM_2.5_ on BAD protein expression

**Fig. 5:**
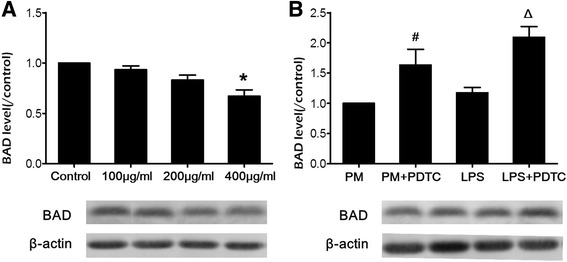
Effects of PM_2.5_ and an NF-κB activator/inhibitor on BAD protein expression. **a**. Cells were treated with 100, 200, and 400 μg/ml PM_2.5_. **b**. Cells were treated with 200 μg/ml PM_2.5_ and an NF-κB activator (100 ng/ml LPS) or inhibitor (100 μmol/L PDTC). CHO cells were cultured in 6-well plates at a density of 5.0 × 10^4^ cells/well in 2 ml of F12-K medium supplemented with 10% FBS and incubated overnight. The media was changed, and the cells were treated with test samples for 24 h. Western blotting was performed. Each blot of BAD protein was reprobed for β-actin as a loading control. Values are expressed as the mean ± SD. Each data point represents an average of three samples. Compared with the untreated control, ^*^ *P* < 0.05; compared with PM_2.5_ 200 μg/ml, ^#^ *P* < 0.05; compared with LPS, ^Δ^ *P* < 0.05

Bcl-xl/BAD is a pro-apoptotic protein in the Bcl-2 family. BAD promotes apoptosis in response to caspases. We used Western blotting to compare the BAD protein levels in the presence of different concentrations of PM_2.5_, as shown in Fig. 5. Compared to the untreated control cells, 100 and 200 μg/ml PM_2.5_ decreased BAD protein expression by 0.93- and 0.83-fold, respectively. BAD protein expression decreased after treatment with 400 μg/ml PM_2.5_ by 0.67-fold (*P* < 0.05). Compared to the 200 μg/ml PM_2.5_ group, the BAD protein expression for the treatment group exposed to 200 μg/ml PM_2.5_ and 100 μmol/L PDTC significantly increased with a fold-change of 1.63 (*P* < 0.05). Compared to the LPS group, the BAD protein expression in the group exposed to 100 ng/ml LPS and 100 μmol/L PDTC increased significantly, by 1.77-fold (*P* < 0.05).

## Discussion

Although many studies have investigated the toxicity of PM_2.5_ on airway cells and other cell types [33–35], the biological mechanisms remain unclear. Therefore, we investigated the effects of PM_2.5_ on the NF-κB pathway in cell apoptosis to understand the implications of PM_2.5_ in Beijing’s air.

The cell viability rate was 72.28% at 200 μg/ml of PM_2.5_ in our study, and this concentration level was used as the main exposure concentration for testing other endpoints. This concentration was higher than previous studies that used total particulate matter (TPM) from mainstream cigarette smoke with CHO cell lines [36–38]. Cigarette smoke is a dynamic aerosol composed of more than 4000 chemical components. The main components include nicotine, alkaloids, tar, CO, polycyclic aromatic hydrocarbons, hydroxyl compounds, and heavy metals [39, 40]. Particle matter is a complex mixture with a physical nucleus that is composed of elemental carbon, nitrates, sulfates, various metals and organic compounds [41]. The major sources of PM_2.5_ in our study were residential and commercial emissions. Fly ash particles were the main components of the PM_2.5_ due to the sampling date, which was during the winter heating season. Compared to cigarette smoke, the content of hazardous substances in our air particles was relatively reduced, so the cytotoxicity was lower than that of cigarette smoke with the same treatment concentration in CHO cells.

There are many methods for detecting the activation of NF-κB, including an IKK assay to detect the degradation of IκB [42], an immunocytochemical method to measure nuclear translocation of NF-κB p65 [43], an electrophoretic mobility shift assay (EMSA) to determine NF-κB-DNA binding activity [25], western blot analysis to detect the NF-κB-regulated protein expression [44], and RT-PCR to detect the mRNA expression [45]. However, these techniques have limitations, and the greatest limitation is that detection is fixed at specific time points (e.g., 1 h, 4 h); thus, it is difficult to capture the dynamic activation of NF-κB. In our study, we used the high content live cell image analysis system IN Cell Analyzer 1000 to investigate NF-κB activity continuously and dynamically. IN Cell Analyzer 1000 is an automated cellular and subcellular imaging system for fast, automated imaging at multiple wavelengths and analysis in fixed and live cells. The IN Cell Investigator software suite provides a comprehensive solution to analyze a wide range of cellular signaling responses. Based on our survey, we think our study is the first to explore NF-κB activity continuously with a live cell image analysis system.

Our results show that the activation of NF-κB increased after exposure to 200 μg/ml PM_2.5_, and the average ratios of NF-κB activation at 24 h were higher than the control group. Our results show that PM_2.5_ increases the activation levels of NF-κB, which is consistent with previous studies of PM_2.5_-induced NF-κB activation [46, 47]. We also observed an activation peak in NF-κB activation at different PM_2.5_ concentrations. With treatments of 100, 200 and 400 μg/ml PM_2.5_ in Fig. 3b, the activation peak of the 200 μg/ml group was the highest, and there was no obvious dose-dependent effect. This result may have occurred because the highest dose treatment of PM_2.5_ induced the highest cytotoxicity, which affects the process associated with green fluorescent protein expression. The peaks for 200 and 400 μg/ml PM_2.5_ appeared after 13.5 h of PM_2.5_ exposure, whereas the peak value of LPS occurred after 5.5 h. These results indicate that the launch time of NF-κB activation is associated with the activator type rather than with the concentration. This is the first analysis of NF-κB activation using a dynamic method. These findings further our understanding of the NF-κB pathway and may help in the development of a new strategy using a NF-κB molecular target.

Whether atmospheric particulate matter induces and leads to persistent NF-κB activation is not clear. Our results show that PM_2.5_-induced NF-κB activation is persistent up to 24 h. Similarly, a previous report on NF-κB activation induced by cigarette smoke indicates that NF-κB activation is persistent for 24 h in human non-small cell lung carcinoma [48].

Cell survival or apoptosis is regulated by the interaction between pro-apoptotic and anti-apoptotic factors, and the normal and quantitative balance of both factors is very important for maintaining the normal physiological functions of a cell [49]. In normal cells, cell death is a programmed process and is affected by many factors. The CHO cells are Chinese hamster ovary cells that are normal growth cells, not tumor cells. In our study, we found that pro-apoptotic BAD protein expression decreased after treatment with PM_2.5_. This result indicates that normal cells have a stress response to external damage factors with a self-protection mechanism [50]. This self-protection mechanism makes changes to the original apoptosis to resist the external damage by strengthening anti-apoptotic factors while the pro-apoptosis response weakens, which arises as a decrease in expression of the pro-apoptotic BAD protein. Compared to persistent and hazardous external damage factors, the cell’s self-protection is still too weak and ultimately leads to cell apoptosis.

BAD is a promoter of apoptosis in the Bcl-2 family that promotes apoptosis and interacts with caspases. A previous study showed that the inhibition of the Akt/PKB signaling pathway activation by β-carotene results in the decreased phosphorylation of BAD protein [51]. Akt-knockout liver cancer cells showed lower p-Bad expression and higher Bad expression, which reduces Bcl-xL expression and promotes down-stream signals, such as cytochrome c, Apaf-1, caspase-9 and caspase-3 expression, and it subsequently contributes to apoptosis in cancer cells [27]. The activation of IKK by Akt causes phosphorylation and degradation of I-κB, which leads to localization of NF-kB to the nucleus, where it can induce the transcription of antiapoptotic genes [52]. It seems that Akt is an important upstream element of the NF-κB pathway and that the BAD protein might impact inflammatory responses and apoptosis. In our study, we found that BAD protein expression decreased after treatment with PM_2.5_. These findings suggest that modulation of the NF-κB activity and its dependent apoptotic regulators are involved in PM_2.5_-induced apoptosis.

In combination with the experimental results obtained in this study, we made a preliminary conclusion that the toxicological mechanism of PM_2.5_ involved the particle matter activating the NF-κB signaling pathway continuously, which promotes the expression of apoptotic genes and proteins and causes cell apoptosis.

## Conclusions

In summary, PM_2.5_ induces NF-κB activation, which persists for 24 h. The expression of the pro-apoptotic protein BAD decreased with increased concentrations of PM_2.5_. Therefore, PM_2.5_ plays a major role in apoptosis by activating the NF-κB signaling pathway and reducing BAD protein expression. Further exploration of the role of NF-κB in apoptosis will provide a scientific basis for atmospheric pollution countermeasures and for further studies of disease prevention.

## Abbreviations

BAD: Bcl-2-associated death promoter
CHO: Chinese hamster ovary
d2EGFP: Destabilized enhanced green fluorescent protein
DMSO: Dimethyl sulfoxide
EMSA: Electrophorectic mobility shift assay
FBS: Fetal bovine serum
IκB: Inhibitor of κB
LPS: Lipopolysaccharide
NF-κB: Nuclear factor-kappa B
PBS: Phosphate-buffered saline
PDTC: Pyrrolidine dithiocarbamate
PM: Particulate matter
PMSF: Phenylmethylsulfonyl fluoride
